# Global Research Trends and Future Directions for Buckwheat as a Smart Crop: A Bibliometric and Content Analysis

**DOI:** 10.3390/foods13244068

**Published:** 2024-12-17

**Authors:** Yongbin Gao, Hanghang Hou, Quzhen Cang, Zhuoma Sangdan, Guan Zhao, Yuhao Yuan, Baili Feng

**Affiliations:** 1 College of Agriculture, Northwest A & F University, Xianyang 712100, China; gyb9797@163.com (Y.G.); houhanghang@nwafu.edu.cn (H.H.); 2 Motuo County Dexing Township Agriculture and Animal Husbandry Integrated Service Center, Linzhi 860700, China; 13549043421@163.com (Q.C.); 18089042880@163.com (Z.S.); 3 Tibet Academy of Agricultural and Animal Husbandry Sciences, Lhasa 851418, China; zhaoguan1112@163.com; 4 College of Agriculture, Henan Agricultural University Zhengzhou, Zhengzhou 450002, China

**Keywords:** buckwheat, bibliometrics, visualization analysis, hotspots, research trends

## Abstract

Buckwheat (*Fagopyrum esculentum* Moench) originates from Central Asia and is widely distributed around the world. It is recognized as a versatile food crop due to its nutritional richness. Conducting a systematic analysis of the literature on buckwheat research can help scientific researchers achieve a better understanding of the current state, hotspots, and trends in this field, thereby promoting the sustainable development of buckwheat. The study retrieved a total of 4512 articles related to buckwheat from the Web of Science Core Collection (WoSCC), involving 104 countries (regions), 3220 institutions, and 12,840 authors. The number of research papers on buckwheat is gradually increasing. China, Japan, Poland, the United States, and South Korea were the top five countries in terms of publication volume in this field. Among the top 10 institutions in terms of publication volume, Chinese institutions account for 60%. Northwest A & F University held a leading position in the number of papers published and cited. Research on buckwheat shows that both domestic and international research institutions tend to collaborate more with institutions within their own countries. A comprehensive analysis of journals with a high number of publications and citations in buckwheat research indicated that studies primarily focus on its use as food and its active substances. Analysis of the authors and cited authors indicated that Wu Qi and Zhu F, among others, have high reputations and significant influence in this field. Reference analysis has determined that early research primarily focused on buckwheat as a pseudo-cereal food; mid-term research mainly concentrated on its active substances and cultivation; later research became more comprehensive, focusing on its potential in food, biotechnology, and medical health, which gradually emerged as trends and hot topics. Keyword analysis indicates that buckwheat flour, antioxidant activity, protective biological control, and buckwheat husk are current research hotspots. This study systematically summarizes the current status of research in the field, identifies research hotspots and trends, and provides a reference for future investigations into buckwheat.

## 1. Introduction

Buckwheat originated in Central Asia and is widely distributed across the world, with major cultivation in countries such as Russia, Australia, and India [1,2]. In China, it is primarily cultivated in provinces such as Shaanxi, Gansu, Ningxia, Inner Mongolia, Tibet, and Guizhou [3,4]. Buckwheat typically grows in temperate biomes and is commonly found in wastelands and roadsides, as well as in cultivated fields, foothills, and wilderness [5]. It prefers cold, moist environments [6], has a short growth cycle [7], and is resistant to poor soil conditions [8], thus playing an important role in disaster relief and marginal land utilization [9,10].

As a nourishment grain, buckwheat provides a rich source of macronutrients such as carbohydrates (mainly starch), high-quality proteins, and essential amino acids such as lysine, which is typically deficient in most other cereals [11,12,13]. Being naturally gluten-free, buckwheat serves as an excellent dietary choice for individuals with celiac disease or gluten intolerance [12,14,15]. Rich in flavonoids, vitamins, minerals, chlorophyll, vitamin P, and other nutrients, buckwheat is considered a natural food resource integrating nutrition, health care, and diet therapy [16,17,18]. Rutin, a flavonoid uniquely found in buckwheat, has demonstrated the ability to enhance capillary resistance and mitigate oxidative stress by modulating antioxidant enzyme activity [19,20]. Similarly, quercetin participates in anti-inflammatory pathways by suppressing NF-κB signaling and enhancing mitochondrial function [21,22]. Additionally, buckwheat has a wide range of uses; it can be processed into various foods, such as buckwheat cakes, flour, and tea, and can also be used as a vegetable [23,24,25]. It is also an excellent feed concentrate for livestock such as sheep, cattle, and pigs [3]. The stems, leaves, and seeds of buckwheat can be used in traditional Chinese medicine [25,26]. According to Chinese pharmacopeia records, it is primarily used to treat indigestion, qi deficiency, dysentery, and other ailments [27,28]. Moreover, buckwheat has decontaminating properties, and in Japan, it is used for washing hair and clothes [29]. The shell of buckwheat is not only a raw material for extracting potassium carbonate but also serves as a good fuel [30,31,32].

Due to the labor-intensive management and low economic returns, buckwheat has long been regarded as a minor grain crop in many countries [33]. Currently, global buckwheat production faces several challenges, including poor production conditions, extensive management practices, low mechanization, difficulties in variety improvement, and outdated breeding methods [34,35]. The specific characteristics of buckwheat production and the weak research foundation have caused issues in Chinese buckwheat cultivation, including poor production conditions, extensive management, low yields, unsuitability for mechanized farming, and challenges in variety improvement [36,37,38]. As living standards rise and scientific dietary concepts become more widespread, coupled with the trend toward mechanized and simplified cultivation and the growing demand for high-quality and diverse products, the demand for buckwheat continues to increase [36,39]. However, the breeding of buckwheat varieties started late and has significantly lagged behind that of other major crops [40]. Therefore, much work remains to be done in improving yield, quality, stress resistance, efficiency, and mechanized production [36,41].

Bibliometric analysis originates from the quantitative study of scientific literature, aiming to reveal inherent patterns and trends in scientific development through statistical and mathematical methods [42]. With the rapid development of science and technology and the exponential growth of information, bibliometric analysis plays an increasingly significant role in scientific research [43]. Through bibliometric analysis, a systematic review, evaluation, and analysis of research achievements in a given field can be conducted, enabling an understanding of the contributions and influence of different scholars and institutions [44]. This facilitates the construction of an academic network and knowledge map, which can reveal the hotspots, frontiers, and developmental trends in the research area [45]. Bibliometric analysis has been conducted in various fields, such as plant agronomic characteristics [46], soil nutrient research [47], plant microbial fuel cells [48], and endangered plants [49]. Bibliometric studies in agronomy have made significant progress in identifying global research trends and influential areas [50]. These analyses help uncover key topics, such as sustainability, crop technologies, and environmental impacts, while providing insights into the productivity and collaboration patterns within the field [51,52]. For instance, studies such as those conducted by Salmerón-Manzano and Manzano-Agugliaro [53] highlight how bibliometric tools can trace trends related to agronomic research and sustainability practices.

Through a review of the relevant literature on buckwheat research, it was found that no prior studies have conducted a bibliometric analysis of buckwheat. By conducting the first bibliometric analysis of buckwheat research, this study fills a historical gap in the field. Using a comprehensive keyword approach, this study explores various aspects of buckwheat research and, in combination with research hotspots and global trends, identifies future directions [54,55]. By combining bibliometric analysis with content analysis and applying visualization tools such as VOSviewer and dual-mapping methods, this study enhances both the depth and comprehensiveness of the analysis while improving the visualization of research results, thereby supporting a deeper understanding of buckwheat research [56,57,58]. Specifically, this study aims to address the following three core questions:

1. What is the distribution of publications and research work on buckwheat over the past two to four years?
2. What are the hot topics in buckwheat research over the past 24 years?
3. What should be done in the next phase of buckwheat research?

Based on this, this article systematically analyzes the current status, research hotspots, and cutting-edge trends in the field of buckwheat research and proposes a series of recommendations for future research and development, aiming to provide scientific guidance and references for researchers in this field.

## 2. Materials and Methods

### 2.1. Data Collection and Retrieval Strategies

The WoSCC database is widely regarded as the optimal resource for literature analysis owing to its highly accurate classification of literature types, extensive scope of application, minimal usage limitations, robust support for cross-comparisons, and the facilitation of advanced assessments based on the data collected [59,60]. This study conducted a search in the WoSCC database on 28 September 2024, to identify the literature related to buckwheat research published between 2000 and 28 September 2024. The search strategy employed TS = (“buckwheat” OR “*Fagopyrum esculentum*”), and the search was restricted to literature types classified “article” and “review”. For data retrieval, two researchers independently reviewed the papers by reading their abstracts. In cases of disagreement, a third researcher was consulted to resolve the discrepancies. The results were then summarized and integrated for subsequent analysis. The retrieved literature was screened based on the following inclusion criteria: (1) full-text publications related to buckwheat, and (2) articles and review manuscripts written in English. The exclusion criteria were: (1) topics unrelated to buckwheat, and (2) conference abstracts, news items, briefings, and similar publications [61,62]. Finally, the plain text versions of the papers were exported for further analysis.

### 2.2. Data Extraction, Analysis and Visualization

VOSviewer (v. 1.6.18) is a free, Java-based software developed by Waltman et al. in 2009, designed to process large datasets and visualize them in a map format [63,64]. CiteSpace (v. 6.2.4 R), created by Professor Chaomei Chen, employs an experimental framework to explore emerging concepts and assess established technologies [65,66]. This software generates bibliographic coupling network diagrams to visualize research frontiers and trends, as well as to forecast potential future research developments [67]. GraphPad Prism (v. 8.0.2) was applied to analyze and visualize trends in annual publication volumes and national publication contributions. Both CiteSpace (v. 6.2.4R, 64-bit advanced version) and VOSviewer (v. 1.6.18) were utilized for data analysis and the construction of scientific knowledge maps.

## 3. Results

### 3.1. Literature Quantity Analysis

**Figure 1:**
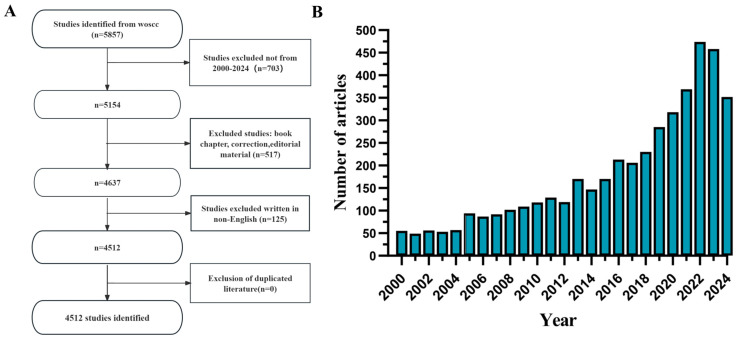
Statistical chart of the number of published papers ((**A**) Screening and results of effective number of papers; (**B**) Annual volume of publications).

The preliminary screening results showed that by using the search terms TS = (“buckwheat” OR “*Fagopyrum esculentum*”) in WoSCC, a total of 5857 documents were retrieved, and 121 studies unrelated to buckwheat. After applying the selection criteria, 703 documents published outside the years 2000 to 2024, along with 517 book chapters, editorial materials, and 125 non-English studies, were excluded, leaving 4512 valid documents (Figure 1A). These documents comprise 4268 articles and 244 reviews, covering 104 countries (regions), 3220 institutions, and 12,840 authors.

Since 2000, the number of research papers on buckwheat has shown a general increasing trend over time (Figure 1B). The growth rate of publications can be divided into three stages: the first stage, from 2000 to 2004, was characterized by slow growth, with annual publications below 80, indicating relatively slow development in the field. The second stage saw a rapid increase in publications from 2005 to 2017, followed by a third stage of further growth after 2018, peaking in 2022.

### 3.2. Country and Institutional Analysis

**Figure 2:**
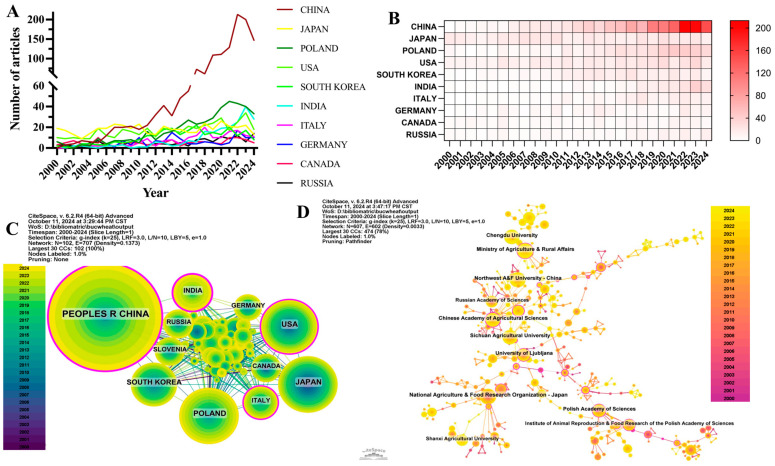
Country and institutional analysis ((**A**) Line graph of national publications; (**B**) Heat map of national publications; (**C**) Networks of country cooperation; (**D**) Networks of institutional cooperation. Different circles represent individual countries or institutions. Different color gradients indicate a time span from 2000 to 2024. The size of each circle corresponds to the number of publications, with larger circles representing a greater volume of publications. Pink borders around certain circles indicate that the node has higher centrality (centrality > 0.1), suggesting that these countries or institutions play a more central role in the network).

An analysis of literature sources and publishing institutions reveals that over the past 24 years, buckwheat research has been conducted by 3220 institutions across 104 countries or regions. Based on annual publication volumes (Figure 2A,B), the leading five countries in this research area are China, Japan, Poland, the United States, and South Korea. Notably, China contributes 30.76% of the total publications, significantly outpacing other countries.

Among the top 10 countries or regions by publication volume, Chinese papers were cited a total of 27,902 times as shown in Table 1, a figure that surpasses all other countries or regions. However, China’s citation-to-publication ratio (20.10) ranked eighth, reflecting a relatively lower average quality of its publications. Japan, which ranked second in publication volume (455 articles), had the third-highest number of citations (13,503), and its citation-to-publication ratio (29.68) was the highest among all countries, indicating a consistently high standard of its research output. The collaboration network reveals that Japan maintains strong research partnerships with the United States, Italy, and Poland, whereas China shows closer collaboration with Russia, South Korea, and India (Figure 2C). In addition to leading in publication volume and citation frequency, China demonstrated a centrality value of 0.23, highlighting its influential role in this research domain.

Analyzing the 3220 publishing institutions, the top 10 institutions with the highest number of publications were primarily from China (Supplementary Table S1, Figure 2D), accounting for 60%. The National Agriculture and Food Research Organization in Japan published the most papers (139), with 3001 citations and 21.59 citations per paper. The second-ranked institution is the Ministry of Agriculture and Rural Affairs of China, which published 127 papers and was cited 2053 times, with an average of 16.17 citations per paper. Northwest A&F University in China ranked third, with 122 papers, cited 3101 times, averaging 12.45 citations per paper. Chengdu University in China ranked fourth with 121 papers, 1506 citations, and 12.45 citations per paper. Further analysis indicates that both domestic and international institutions predominantly collaborate with partners from their respective countries. It is recommended to strengthen inter-institutional collaboration at both domestic and international levels to address and overcome existing academic barriers.

### 3.3. Journal Analysis

In Table 2, the top 10 journals with the highest output and citation frequency in buckwheat research are listed. *Food Chemistry*, with 198 articles, was the most frequently published journal in buckwheat research, followed by the *Journal of Agricultural and Food Chemistry* (134 articles), *Foods* (118 articles), and *LWT-Food Science and Technology* (94 articles). Among the top 10 most productive journals, *Food Chemistry* had the highest impact factor (IF) of 8.5, and all journals were classified as Q1 or Q2.

**Figure 3:**
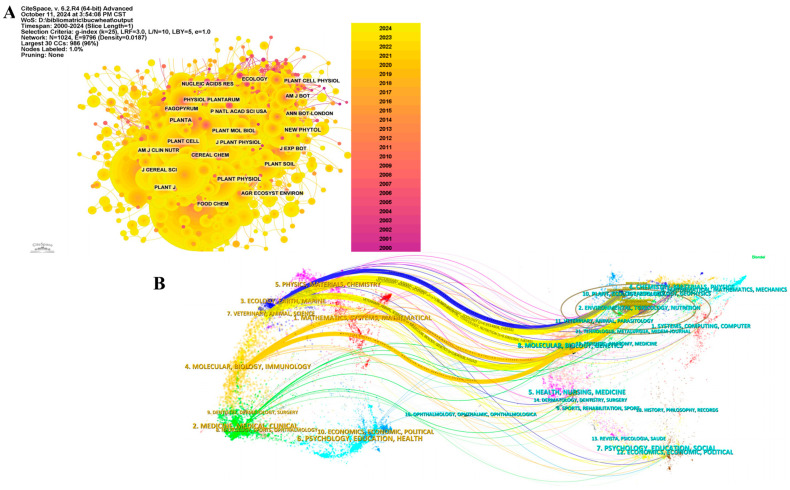
Journal analysis ((**A**) Co-citation network map of journals; (**B**) Dual map of journals).

The influence of a journal is assessed through its co-citation frequency, which reflects its impact within the scientific community [68]. The analysis results (Figure 3A, Table 2) indicate that the journal with the most co-citations is *Food Chemistry* (2340 times), followed by *J Agr Food Chem* (2335 times) and *Food Res Int* (1477 times). Among the top 10 journals with the highest co-citation counts, *Trends Food Sci Tech* had the highest IF of 15.1 and was co-cited 787 times. Among the top 10 journals ranked by co-citation counts, the majority (90%) are categorized as Q1 or Q2.

The thematic distribution of academic publications is illustrated using a dual-map overlay (Figure 3B), which displays citing journals on the left and cited journals on the right, connected by colored paths representing citation links. Three prominent citation paths were identified. Research published in journals in the plant/ecology/zoology fields was primarily cited by research published in journals in the ecology/earth/marine fields. Research published in journals in the plant/ecology/zoology and environment/toxicology/nutrition, and molecular/biology/genetics fields was primarily cited by research published in veterinary/animal/science journals. Research published in the environment/toxicology/nutrition and molecular/biology/genetics fields was primarily cited by research in molecular/biology/immunology journals.

### 3.4. Author and Co-Citation Author Analysis

**Figure 4:**
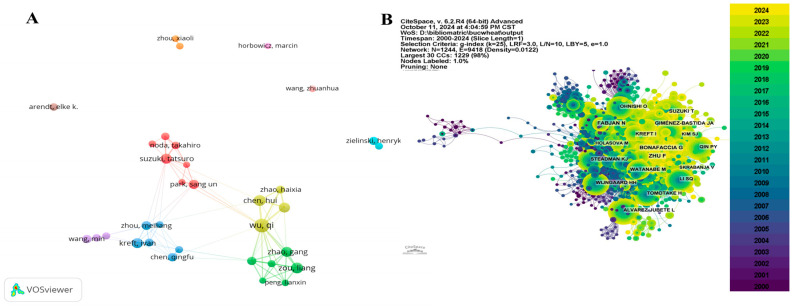
Authors analysis ((**A**) Cooperation network of authors; (**B**) Co-citation network of authors).

Among all the authors contributing to buckwheat research, Table 3 lists the 10 authors with the highest publication counts. The top 10 authors collectively published 527 papers, accounting for 11.68% of the total in this field. Wu Qi published the most papers (91), followed by Zou Liang (60), Chen Hui (58), and Zhao Gang (52). CiteSpace visualizes the collaborative network between authors (Figure 4A). It indicates that Wu Qi’s team (green cluster) and Noda Takahiro’s team (red cluster) are the primary collaborative groups, contributing significantly to buckwheat research. Some transnational and interdisciplinary collaborations are evident among different research groups, particularly between Wu Qi’s group and others. Authors such as Zhou Xiaoli, Arendt Elke K., and Wang Zhuanhua appear to focus more on individual research and exhibit relatively fewer collaborations.

The citation analysis shows that 121 authors have been cited over 50 times, reflecting the high reputation and influence of their work. The top 10 authors with the most co-citations and overall citations were identified from the analysis (Figure 4B and Table 3). The largest nodes correspond to the authors with the most co-citations, such as Zhu, F (412 citations), Bonafaccia, G (382 citations), and Fabjan, N (333 citations).

### 3.5. Co-Citation Reference Analysis

#### 3.5.1. Highly Cited Documents Analysis

**Figure 5:**
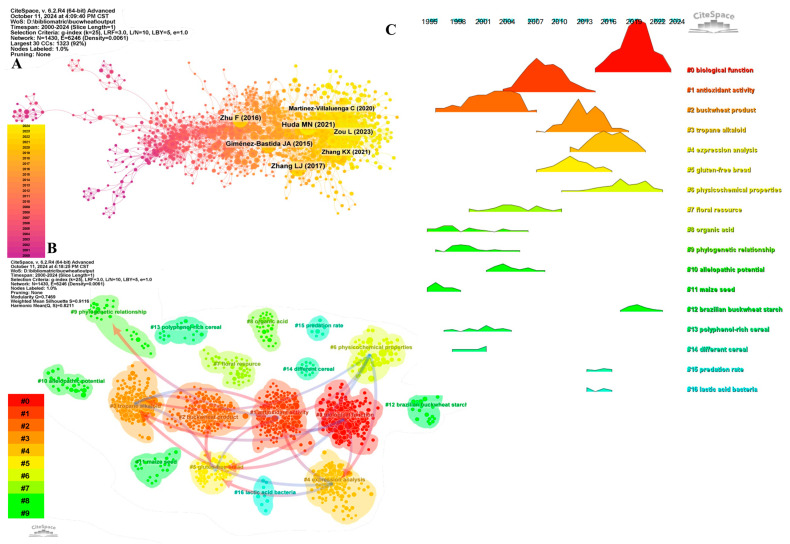
Co-citation reference analysis ((**A**) Co-cited network of literature; (**B**) Clustering of co-cited literature; (**C**) Peak map of co-cited literature).

Using one year as the time slice, the time period from 2000 to 2024 resulted in a citation reference network consisting of 1430 nodes and 6246 links (Figure 5A). The top 10 articles with the highest co-citations were identified (Supplementary Table S2), addressing key research areas such as buckwheat bioactive compounds, nutritional components, genome analysis, environmental stress tolerance, chemical composition, functional food applications, health benefits, protection against metabolic diseases, food development, evolutionary history, gene mining, bioactive substance metabolism, functional tea trends, and multi-omics applications in variety improvement and nutritional product development.

The highest-ranked article, “Treasure from the Garden: Bioactive Compounds of Buckwheat”, published in *Food Chemistry* by Huda, Md. Nurul, reviews recent research on the phytochemicals of buckwheat. The article emphasizes the unique functions and health benefits of bioactive compounds in buckwheat, a gluten-free crop from the Polygonaceae family. Rich in beneficial phytochemicals, buckwheat offers significant health advantages and has adapted to diverse ecological regions worldwide. In recent years, buckwheat has gained popularity as a nutritious, low-calorie health food. Buckwheat’s bioactive compounds include flavonoids (rutin, quercetin, hibifolin, isoquercitrin, vitexin, and isovitexin), fatty acids, polysaccharides, proteins, amino acids, iminosugars, dietary fiber, fucoidan, resistant starch, vitamins, and minerals. These compounds contribute to the exceptional nutritional value of buckwheat. Additionally, some bioactive factors in buckwheat have long garnered attention due to their benefits in treating and preventing various human diseases. The second-ranked article, “The Tartary Buckwheat Genome Provides Insights into Rutin Biosynthesis and Abiotic Stress Tolerance”, authored by Zhang Lijun, details the high-quality, chromosome-level genome sequencing and assembly of the Tartary buckwheat variety Pinku1. The study annotated genes using gene expression evidence and identified a whole-genome duplication (WGD) event that occurred after divergence from beet. Leveraging the assembled genome as a reference, the research reanalyzed an RNA-seq dataset, discovering several novel genes associated with aluminum tolerance. Additionally, comparative genomics and gene expression analysis revealed genes encoding enzymes involved in quercetin biosynthesis, along with MYB transcription factors essential for quercetin synthesis and stress response. Comparative genomic studies further suggested expansions in specific gene families within Tartary buckwheat, which likely contribute to its exceptional aluminum tolerance and resilience to drought and cold stress. The third-ranked article, “Chemical Composition and Health Effects of Tartary Buckwheat”, reviews existing research on the chemical composition and biological functions of Tartary buckwheat, based on in vitro and in vivo studies. The article proposes that Tartary buckwheat has strong potential as a sustainable crop for functional food production, offering considerable health benefits.

#### 3.5.2. Co-Citation Reference Clustering and Temporal Clustering Analysis

Co-citation reference clustering and temporal clustering analyses were performed (Figure 5B,C). The results showed that buckwheat products (cluster 2), organic acids (cluster 8), phylogenetic relationships (cluster 9), maize seed (cluster 11), polyphenol-rich cereals (cluster 13), and various cereals (cluster 14) were early research hotspots. Antioxidant activity (cluster 1), floral resources (cluster 7), and allelopathic potential (cluster 10) represented mid-term research hotspots. Biological functions (cluster 0), tropane alkaloids (cluster 3), expression analysis (cluster 4), gluten-free bread (cluster 5), physicochemical properties (cluster 6), Brazilian buckwheat starch (cluster 12), predation rates (cluster 15), and lactic acid bacteria (cluster 16) represent current hot topics and emerging trends in the field.

### 3.6. Keywords Analysis

**Figure 6:**
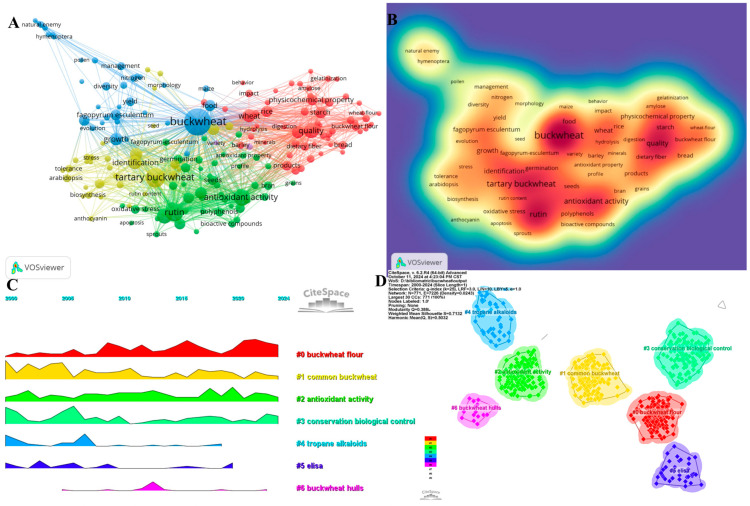
Keywords analysis ((**A**) Network map of high-frequency keywords; (**B**) Density map of keywords; (**C**) Peak map of keyword clustering; (**D**) clustering map of keywords).

Analyzing keywords provides a quick understanding of the current state and development trends in a given field [69]. Based on the keyword co-occurrence analysis in VOSviewer, the most frequently occurring keywords are buckwheat (1176), followed by rutin (489), antioxidant activity (424), quality (358), and flavonoids (347) (Figure 6A,B, Supplementary Table S3). After filtering out irrelevant keywords, a network of 179 keywords that appeared at least 35 times was constructed, resulting in six distinct clusters. The first cluster (red) contains 60 keywords, including quality, digestion, amaranth, baking, bread, wheat, dough, fermentation, fiber, foods, gelatinization, hydrolysis, impact, performance, quinoa, rheology, sensory property, storage, and stability. The second cluster (green) contains 49 keywords, such as rutin, antioxidants, acids, seeds, gut microbiota, capacity, extracts, fractions, health, apoptosis, components, diet, flavonoids, functional food, grains, honey, inhibition, metabolism, models, obesity, profiles, rats, and oxidative stress. The third cluster (blue) includes 35 keywords, such as buckwheat, growth, yield, morphology, diversity, evolution, plants, population, biomass, allelopathy, cover crops, Fagopyrum, food, longevity, maize, natural enemies, nectar, nutrition, pollen, and soil. The fourth cluster (yellow) contains 33 keywords, including allergen, accumulation, anthocyanins, cloning, food allergy, identification, leaves, mechanisms, proteins, purification, resistance, responses, seeds, stress, Tartary buckwheat, toxicity, and transcriptome. The fifth cluster (purple) contains 18 keywords, including grain and variety. Using CiteSpace to visualize research hotspots over time through a volcano plot (Figure 6C,D), it was found that buckwheat flour, common buckwheat, antioxidant activity, conservation biological control, and buckwheat hull are current research hotspots. Experimental studies indicate that buckwheat extract exhibits antioxidant activity exceeding 80%, as measured by DPPH and ABTS assays, at concentrations as low as 50 µg/mL. This remarkable efficacy surpasses that of many common cereal grains. The high levels of rutin (200–400 mg per 100 g) and catechins in buckwheat are likely responsible for this effect, emphasizing its potential to mitigate oxidative stress and maintain cellular homeostasis.

### 3.7. Keyword and Cited Literature Burst Analysis

#### 3.7.1. Cited Reference Burst Analysis

Using CiteSpace, 50 of the most significant citation bursts related to buckwheat research were identified. The focus primarily lies in the study of buckwheat as a pseudo-cereal crop, examining its nutritional and bioactive compounds, health benefits to humans, functional food development, and research on buckwheat cultivation and molecular aspects.

The top-ranked article, “Chemical Composition and Health Effects of Tartary Buckwheat”, published in *Food Chemistry*, provides a review of the current understanding of Tartary buckwheat’s chemical composition and biological functions, based on in vitro and in vivo models. It proposes that Tartary buckwheat holds significant potential for further development as a sustainable crop for functional food production, contributing to improved human health.

The second-ranked article, “Buckwheat as a Functional Food and Its Effects on Health”, published in the *Journal of Agricultural and Food Chemistry*, reviews recent advancements in understanding the health benefits of buckwheat. Drawing on in vitro and in vivo studies, the article emphasizes the specific effects of buckwheat’s bioactive compounds and explores the underlying mechanisms behind these benefits.

The third-ranked article, “The Tartary Buckwheat Genome Provides Insights into Rutin Biosynthesis and Abiotic Stress Tolerance”, published in *Molecular Plant*, investigates Tartary buckwheat (Fagopyrum tataricum) as a significant pseudo-cereal crop adapted to harsh environmental conditions. The study presents the assembly of a high-quality, chromosome-level genome for Tartary buckwheat and underscores its significance by identifying genes involved in the rutin biosynthesis pathway, along with MYB transcription factors that regulate this process. Furthermore, leveraging RNA-seq datasets and existing drought and cold tolerance studies, the research identifies a range of novel aluminum-resistant genes previously unreported.

**Figure 7:**
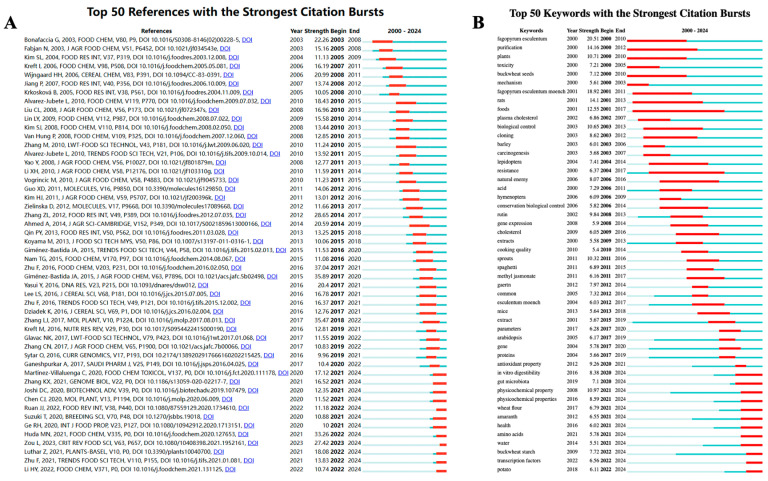
Co-cited references and keyword burst analysis ((**A**) Bursting map of cited literature; (**B**) Bursting map of keywords).

The 50 emerging references were all published from 2000 to 2024, demonstrating their frequent citation over the past two decades. Notably, 12 of these papers are currently at their citation peak (Figure 7A), suggesting that research related to buckwheat will likely continue to attract significant attention in the future.

#### 3.7.2. Keyword Burst Analysis

Among the 675 emerging keywords in this field, attention was directed toward the top 50 with the strongest emergence (Figure 7B), representing the current research hotspots and potential future research directions. The analysis of these keywords leads to the following conclusions:

In the fields of agriculture and food, current research on buckwheat spans several areas, including:

Agriculture: Keywords such as “bitter buckwheat” and “buckwheat varieties” reflect research on different types of buckwheat, including variety breeding and resistance studies. Keywords such as “biological control”, “natural enemies”, and “protective biological control” indicate research on pest and disease management in agriculture. The keyword “plant” reflects the research scope of buckwheat as an agricultural crop.

Food science: Keywords such as “food”, “pasta”, “flour”, and “cooking quality” involve the application of buckwheat in food products and food processing research. “Buckwheat starch” emphasizes the characteristics and applications of buckwheat’s primary component in food. The keyword “water” may relate to its role in the processing of buckwheat food products.

Biochemistry: Keywords such as “purification”, “rutin”, “antioxidant properties”, “amino acids”, “proteins”, and “extracts” reflect the extraction, properties, and functions of various bioactive components in buckwheat. “Physicochemical properties” focuses on the chemical and physical characteristics of buckwheat from a broader perspective. “In vitro digestibility” studies the performance of buckwheat during digestion outside the human body.

Medical and health: Keywords such as “rats” and “mice” indicate the use of animal models to study the effects of buckwheat on health, including “plasma cholesterol”, “intestinal flora”, and “carcinogenicity.” The keyword “health” directly reflects the focus on the relationship between buckwheat and human health.

Biotechnology: Keywords such as “cloning”, “genes”, “gene expression”, and “transcription factors” relate to the study and manipulation of buckwheat genes using biotechnological methods.

### 3.8. Research Progress on Nutritional Components and Functional Properties of Buckwheat

#### 3.8.1. Protein

Buckwheat contains approximately 13% protein, which is significantly higher than most staple crops, such as rice and wheat [22]. Buckwheat protein is renowned for its balanced amino acid composition, particularly its high lysine content, which compensates for the amino acid deficiencies of other cereals [70]. The primary components of buckwheat protein include albumin, globulin, prolamin, and glutelin, with albumin and globulin together accounting for more than 60% of the total protein [71]. This composition results in high digestibility and bioavailability [18].

Recent studies highlight the functional bioactivities of buckwheat protein, including its potential to lower plasma cholesterol levels, improve blood pressure, alleviate intestinal inflammation, and regulate lipid metabolism [72]. Enzymatic hydrolysis enhances the release of bioactive peptides, which exhibit antioxidant, anti-inflammatory, and immunomodulatory properties [18]. Moreover, high-pressure or enzymatic treatments can significantly reduce the allergenicity of buckwheat protein, making it a promising ingredient for hypoallergenic food products [70]. Extraction techniques, such as ultrasound-assisted extraction (UAE), pH regulation, and response surface methodology optimization, have further improved the efficiency and purity of buckwheat protein extraction, facilitating its application in food and functional food industries [71].

#### 3.8.2. Dietary Fiber

Buckwheat dietary fiber is a key nutritional advantage, with a total content of 40–50%, far exceeding that of wheat, rice, and other staple crops [73]. This dietary fiber consists of both soluble fibers (e.g., gums, galacturonic acid) and insoluble fibers (e.g., cellulose, lignin), with insoluble fibers predominating [74]. Insoluble fibers promote intestinal peristalsis and prevent constipation, while soluble fibers delay gastric emptying, slow glucose absorption, and improve insulin sensitivity [75].

Studies have demonstrated that buckwheat dietary fiber exhibits antioxidant, anti-inflammatory, and anti-glycation properties, particularly in preventing metabolic diseases such as diabetes and obesity [76]. Advanced extraction techniques, including enzymatic treatment, high-pressure processing (HPP), and microwave-assisted extraction (MAE), have significantly enhanced the functional properties of dietary fiber, such as water-holding capacity, oil-binding capacity, and solubility [73]. Furthermore, buckwheat dietary fiber positively impacts gut microbiota by promoting the growth of beneficial bacteria such as Lactobacillus and Bifidobacterium, thereby improving host metabolic health [75].

#### 3.8.3. Minerals and Vitamins

Buckwheat is rich in minerals and vitamins, with significantly higher levels compared to rice and wheat [72]. It is particularly abundant in magnesium (Mg), potassium (K), iron (Fe), and zinc (Zn), which are vital for bone health, nerve function, and immune regulation [77]. Selenium (Se) in Tartary buckwheat is especially notable for its potent antioxidant and immune-enhancing properties, as it effectively eliminates free radicals and strengthens the immune system by regulating glutathione peroxidase (GPx) activity [24].

Buckwheat is also an excellent source of vitamins, especially B-complex vitamins (e.g., thiamine, riboflavin, and niacin) and fat-soluble vitamins (e.g., vitamin D and vitamin E) [72]. The synergistic effects of vitamin E and polyphenolic compounds provide significant antioxidant activity, suppress lipid peroxidation, slow aging, and reduce cardiovascular disease risk [77].

#### 3.8.4. Flavonoids

Buckwheat is a rich source of flavonoids, such as rutin, quercetin, and isoquercitrin, which contribute to its broad health benefits [78]. Rutin, one of the most prominent flavonoids in buckwheat, accounts for over 2% of the seeds’ dry weight and plays a key role in reducing the risk of chronic diseases such as atherosclerosis and diabetes [79].

Flavonoids in buckwheat also enhance insulin sensitivity, lower blood glucose levels, and regulate lipid metabolism, thereby mitigating obesity [80]. Furthermore, they inhibit adipocyte differentiation and reduce visceral fat accumulation [81]. Recent research indicates that buckwheat flavonoids regulate the expression of pro-inflammatory factors (e.g., IL-6, TNF-α), suppress inflammatory responses, and offer therapeutic potential for conditions such as arthritis and cardiovascular disorders [81].

## 4. Discussion

### 4.1. Current Status of the Buckwheat Research Field

The annual output of papers in buckwheat research has demonstrated a steady growth trend, with a significant increase starting in 2005, highlighting the growing importance of this research field. Analysis reveals that Wu Qi is the most prolific author, having published 91 papers, ranking first. He also has the highest centrality, reflecting his substantial influence and prominence in the field. Additionally, Zhu F holds the highest number of citations (412). China leads in publication output with 1388 papers, more than triple that of the United States (425), followed by Japan (455), Poland (433), and South Korea (253). China also holds the highest centrality, further reflecting its significant influence and prominence in the field. These disparities in publication output may be attributed to factors such as socioeconomic status, research capacity, and population size. Among the top 10 institutions in publication output, 60% are from China, with Chinese institutions accounting for 4 of the top 5. Chinese researchers and institutions consistently rank at the top, indicating alignment among countries, institutions, and authors with high publication output. However, both domestic and international institutions tend to collaborate primarily within their own countries. Thus, fostering stronger collaboration between domestic and international institutions is crucial for breaking down academic barriers and advancing the field.

Regarding journals, both the highest publication volume and co-citations are attributed to *Food Chemistry*. This journal is highly regarded in food science, covering areas such as the analysis of food components, chemical changes during processing, food additives, and the chemistry of flavor and aroma. This suggests that buckwheat research is primarily focused on its application as a food product.

### 4.2. Hotspots and Trends in Buckwheat Research

Keywords provide a concise summary of an article’s theme and can clearly highlight research hotspots and trends in the relevant field [82]. Keyword clustering timelines and burst analysis assist researchers in better understanding the evolution of buckwheat research topics. The volcano plot generated by CiteSpace visualizes research hotspots over time (Figure 6C,D), indicating that buckwheat flour, common buckwheat, antioxidant activity, protective biological control, and buckwheat products are current research hotspots. Keyword analysis confirms that buckwheat flour, common buckwheat, antioxidant activity, protective biological control, and the development of buckwheat by-products remain key research hotspots. Keyword burst results highlight buckwheat variety cultivation, resistance research, agricultural pest control, food processing applications, characteristics and uses of key components, extraction, properties, and functional research of bioactive compounds, health impacts, and biobreeding as prominent research hotspots and trends.

A comprehensive analysis indicates that as science and technology advance, interest in buckwheat research continues to grow. Research has gradually shifted from basic studies to the use of modern technologies for cultivation, management, and the diversified development of buckwheat. Based on this shift, future research trends in buckwheat are expected to focus on the following areas:

Nutrition and health effects research: Animal studies investigating the impact of buckwheat on plasma cholesterol, intestinal flora, and its potential anti-cancer properties, along with other health benefits. Research on the functions of key nutritional components in buckwheat, including amino acids, proteins, and rutin, also remains a key area of focus [39,83,84,85].

Variety improvement and biological control: Research into buckwheat resistance, new variety cultivation, and biological control methods for pest and disease reduction, aimed at improving yield and quality [23,86].

Food development and processing: Development of buckwheat-based foods such as pasta and flour, alongside studies of cooking quality and processing characteristics, as well as the application of buckwheat’s bioactive components in food [81,87,88].

Biotechnology applications: Employing biotechnologies such as cloning and gene expression to investigate buckwheat gene functions, supporting variety improvement and functional development [89,90,91].

Bioactive component research: Purification and study of the physicochemical and antioxidant properties of bioactive components in buckwheat, such as rutin, to explore their application in medicine and food [87,92,93].

### 4.3. Buckwheat: Demonstrating Multifaceted Potential as an Exceptional Climate-Resilient Crop

Buckwheat demonstrates exceptional adaptability to diverse environmental conditions, making it a valuable crop in agricultural systems addressing climate change [94]. Its ability to thrive in poor soils, arid conditions, and acidic environments highlights its suitability for marginal lands and sustainable farming practices [95]. Additionally, its short growth cycle of 70 to 90 days enhances its flexibility for cultivation, particularly in crop rotation systems or regions with limited growing seasons affected by climate variability [71]. From a nutritional perspective, buckwheat is rich in high-quality proteins, essential amino acids, and antioxidants such as rutin and quercetin, which have been shown to protect against cardiovascular diseases, diabetes, and other chronic conditions [96]. Moreover, as a gluten-free crop, buckwheat is an ideal food source for individuals with gluten intolerance, and its increasing demand in the gluten-free food market underscores its potential for market expansion [81]. Economically, the multifunctionality of buckwheat enhances its value in sustainable agriculture. In addition to serving as food and feed, buckwheat is also a valuable green manure that improves soil structure and fertility, supporting subsequent crop productivity [97]. Its low input requirements and resilience to stress reduce dependency on chemical fertilizers and pesticides, aligning with the principles of climate-smart agriculture [98]. Recent advances in buckwheat genomics have further unlocked its potential for genetic improvement. Modern genomic technologies have identified key genes associated with drought resistance, cold tolerance, and disease resistance, offering targeted avenues for improving its productivity and adaptability [99]. Genome-wide association studies and functional gene identification have accelerated the development of high-yield and nutritionally superior buckwheat varieties [100]. In summary, buckwheat, with its outstanding environmental adaptability, substantial nutritional value, and significant economic potential, represents a critical crop for addressing the challenges of climate change and global food security. Future efforts in genetic improvement, policy support, and market development will further enhance its role in sustainable agricultural systems.

### 4.4. What Should Be Done in Future Buckwheat Research

Based on the findings of this article and a comprehensive review of current domestic and international research on buckwheat, the following future research directions are proposed:

Genetic improvement and variety cultivation: Buckwheat breeding programs lag significantly behind those of other major crops [101]. Future research should prioritize the development of high-yield, stress-resistant varieties through genetic advancements [98,102]. Modern biotechnologies, such as gene editing, cloning, and transcriptomics, can aid in identifying genes that confer tolerance to abiotic stresses (e.g., drought, salinity, and aluminum toxicity), thereby improving buckwheat’s adaptability across diverse environments [23,89,90].

Nutrition and health benefits research: Although extensive research has been conducted on buckwheat’s bioactive compounds (e.g., rutin and quercetin), further studies are required to explore their specific health benefits, particularly their effects on chronic diseases such as cardiovascular diseases, diabetes, and cancer [84,90,93,103]. Animal models and clinical trials should be employed to investigate the therapeutic potential of these bioactive compounds. Additionally, future research should investigate the synergistic effects of buckwheat components in combination with other foods [92,104].

Mechanization and sustainable agriculture: To address the challenge of low mechanization levels in buckwheat production, research should focus on developing farming techniques compatible with mechanized production systems while considering buckwheat’s unique growth cycle and environmental conditions [23,36,105]. Additionally, integrating biological control methods, such as promoting the use of natural pest enemies, can increase yields and reduce reliance on chemical pesticides, thereby supporting more sustainable agricultural practices [86,106].

Functional food and by-product development: With the increasing consumer interest in health-promoting foods, it is essential to explore new buckwheat-based functional food products [107,108]. This includes studying the effects of processing technologies on buckwheat’s nutritional value, flavor, and bioavailability [109,110,111]. Furthermore, the potential applications of buckwheat by-products, such as using husks for bioenergy production or as eco-friendly packaging materials, merit further investigation [112,113].

Environmental impact and climate adaptability: Research on buckwheat’s role in carbon sequestration and its potential as a crop for marginal lands could contribute to global climate change mitigation efforts [38]. Studies on the environmental benefits of buckwheat cultivation, including its positive effects on soil health and biodiversity, will be crucial for promoting its use in sustainable agricultural systems [114,115,116].

By focusing on these research areas, future studies can bridge existing gaps and contribute to the global development and utilization of buckwheat as a valuable crop for nutrition, health, and sustainability.

## 5. Conclusions

This study employed bibliometric methods to evaluate and quantify 4512 papers related to buckwheat research published over the past 24 years. According to this analysis, institutions from China were the most productive, while researchers from developed countries, including the United States and Japan, actively participated in global academic exchanges. Researchers such as Wu Qi and Zhu F have considerable academic influence in this field. The journal that has published the most papers on this topic is *Food Chemistry*. Currently, research in this area primarily focuses on food development and processing, molecular and biotechnological breeding, and the field of medical health. Future research directions are likely to focus on nutritional and health efficacy studies, food development and processing, biotechnological applications, bioactive component research, variety improvement, and biological control. In the future, to fully realize the potential of buckwheat, a multidisciplinary approach is indispensable. Integrating advanced genomic technologies, such as CRISPR, with traditional breeding methods will be pivotal in developing stress-tolerant varieties. Collaborative efforts spanning agronomy, food science, and pharmacology are critical for driving innovation in high-value-added products. Furthermore, policy initiatives designed to incentivize buckwheat cultivation, especially in marginal and underutilized regions, will reinforce its contribution to global food security. By aligning scientific research with market demands and sustainable agricultural practices, buckwheat can establish itself as a cornerstone of resilient and adaptable food systems.

In summary, this article systematically reviews the current state of research in the field of buckwheat, identifies research hotspots and trends, and proposes prospects and feasible suggestions for future research, aiming to provide a reference for subsequent researchers. However, this study has certain limitations. It analyzed literature from only a single database, which may not fully capture the current state of research in the field. Moreover, since the database is updated in real-time, some new papers may not have been included, potentially affecting the analysis results. Moving forward, we will build upon this study, continuously monitor research developments in the field of buckwheat, analyze emerging hotspots and trends in a timely manner, and provide reliable references and insights for agricultural researchers.

## Figures and Tables

**Table 1:** Table of published literature, by country.

Rank	Country/Region	Article Counts	Centrality	Percentage	Citations	Citation Per Publication
1	CHINA	1388	0.23	30.76%	27,902	20.1
2	JAPAN	455	0.08	10.08%	13,503	29.68
3	POLAND	433	0.07	9.60%	8977	20.73
4	USA	425	0.16	9.42%	15,052	35.42
5	SOUTH KOREA	253	0.1	5.61%	6613	26.14
6	INDIA	208	0.2	4.61%	3681	17.7
7	ITALY	169	0.1	3.75%	5875	34.76
8	GERMANY	145	0.08	3.21%	4976	34.32
9	CANADA	138	0.04	3.06%	3792	27.48
10	RUSSIA	135	0.05	2.99%	1527	11.31

Centrality: a measure of a country’s or region’s influence within the global academic network. A higher centrality value indicates greater connectivity and impact on international research collaboration and communication. It reflects the country’s position within the scholarly network, where higher values signify central involvement in global scientific exchanges and collaborations.

**Table 2:** Publications and co-citations by journals.

Rank	Journal	Article Counts	Percentage (4512)	IF	Quartile in Category	Rank	Cited Journal	Co-Citation	IF (2023)	Quartile in Category
1	Food Chemistry	198	4.39%	8.5	Q1	1	FOOD CHEM	2340	8.5	Q1
2	Journal of Agricultural and Food Chemistry	134	2.97%	5.7	Q1	2	J AGR FOOD CHEM	2335	5.7	Q1
3	Foods	118	2.62%	4.7	Q1	3	FOOD RES INT	1477	7	Q1
4	LWT-Food Science and Technology	94	2.08%	6.0	Q1	4	J CEREAL SCI	1107	3.9	Q2
5	Journal of Cereal Science	84	1.86%	3.9	Q2	5	LWT-FOOD SCI TECHNOL	1090	6	Q1
6	Molecules	65	1.44%	4.2	Q2	6	J SCI FOOD AGR	964	3.3	Q1
7	Plants-Basel	59	1.31%	4.0	Q1	7	CEREAL CHEM	891	2.2	Q3
8	International Journal of Biological Macromolecules	57	1.26%	7.7	Q1	8	J FOOD SCI	833	3.2	Q2
9	Food Research International	51	1.13%	7.0	Q1	9	TRENDS FOOD SCI TECH	787	15.1	Q1
10	Agronomy-Basel	48	1.06%	3.3	Q1	10	CRIT REV FOOD SCI	750	7.3	Q1

**Table 3:** Authors’ publications and co-citations.

Rank	Author	Count	Rank	Co-Cited Author	Citation
1	Wu, Qi	91	1	Zhu, F	412
2	Zou, L	60	2	Bonafaccia, G	382
3	Chen, H	58	3	Fabjan, N	333
4	Zhao, G	52	4	Kreft, I	288
5	Suzuki, T	51	5	Gimenez-Bastida, Ja	280
6	Kreft, I	49	6	Suzuki, T	266
7	Li, C	49	7	Alvarez-Jubete, L	240
8	Park, S	42	8	Li, SQ	236
9	Zhou, M	38	9	Steadman, KJ	228
10	Xiang, D	37	10	Watanabe, M	203

## Data Availability

The original contributions presented in the study are included in the article/Supplementary Materials, further inquiries can be directed to the corresponding author.

## Supplementary Materials

The following supporting information can be downloaded at: https://www.mdpi.com/article/10.3390/foods13244068/s1, Table S1. Table of Institutional Published Literature; Table S2. Co-citation table of literatures; Table S3. High Frequency Keyword Table.
